# Recent Advances in Utilizing Lignocellulosic Biomass Materials as Adsorbents for Textile Dye Removal: A Comprehensive Review

**DOI:** 10.3390/polym16172417

**Published:** 2024-08-26

**Authors:** Manisha Yadav, Nagender Singh, Annu, Suhail Ayoub Khan, Chaitany Jayprakash Raorane, Dong Kil Shin

**Affiliations:** 1 Department of Textile and Fibre Engineering, Indian Institute of Technology Delhi, Hauz Khas, New Delhi 110016, India; 2 Department of Fashion and Apparel Engineering, The Technological Institute of Textile and Sciences, Bhiwani 127021, India; 3 Materials Laboratory, School of Mechanical Engineering, Yeungnam University, 280-Daehak-ro, Gyeongsan 38541, Republic of Korea; 4 School of Chemistry and Chemical Engineering, Yangzhou University, Yangzhou 225009, China; 5 IAMFE, School of Chemistry and Materials Science, Nanjing University of Information Science and Technology, Nanjing 210044, China; 6 School of Chemical Engineering, Yeungnam University, 280-Daehak-ro, Gyeongsan 38541, Republic of Korea

**Keywords:** lignocellulosic biomass, textile dyes, wastewater treatment, adsorption, sustainable

## Abstract

This review embarks on a comprehensive journey, exploring the application of lignocellulosic biomass materials as highly effective adsorbents for the removal of textile dyes (cationic and anionic dyes) from wastewater. A literature review and analysis were conducted to identify existing gaps in previous research on the use of lignocellulosic biomass for dye removal. This study investigates the factors and challenges associated with dye removal methods and signifies their uses. The study delves into the pivotal role of several parameters influencing adsorption, such as contact time, pH, concentration, and temperature. It then critically examines the adsorption isotherms, unveiling the equilibrium relationship between adsorbent and dye and shedding light on the mechanisms of their interaction. The adsorption process kinetics are thoroughly investigated, and a detailed examination of the adsorbed rate of dye molecules onto lignocellulosic biomass materials is carried out. This includes a lively discussion of the pseudo-first, pseudo-second, and intra-particle diffusion models. The thermodynamic aspects of the adsorption process are also addressed, elucidating the feasibility and spontaneity of the removal process under various temperature conditions. The paper then dives into desorption studies, providing insights into the regeneration potential of lignocellulosic biomass materials for sustainable reusability. The environmental impact and cost-effectiveness of employing lignocellulosic biomass materials in textiles including Congo Red, Reactive Black 5, Direct Yellow 12, Crystal Violet, Malachite Green, Acid Yellow 99, and others dyes from wastewater treatment are discussed, emphasizing the significance of eco-friendly solutions. In summary, this review brings together a wealth of diverse studies and findings to present a comprehensive overview of lignocellulosic biomass materials as adsorbents for textile cationic and anionic dye removal, encompassing various aspects from influential parameters to kinetics, adsorption isotherms, desorption, and thermodynamics studies. Its scope and other considerations are also discussed along with its benefits. The collective knowledge synthesized in this paper is intended to contribute to the advancement of sustainable and efficient water treatment technologies in the textile industry.

## 1. Introduction

**Figure 1:**
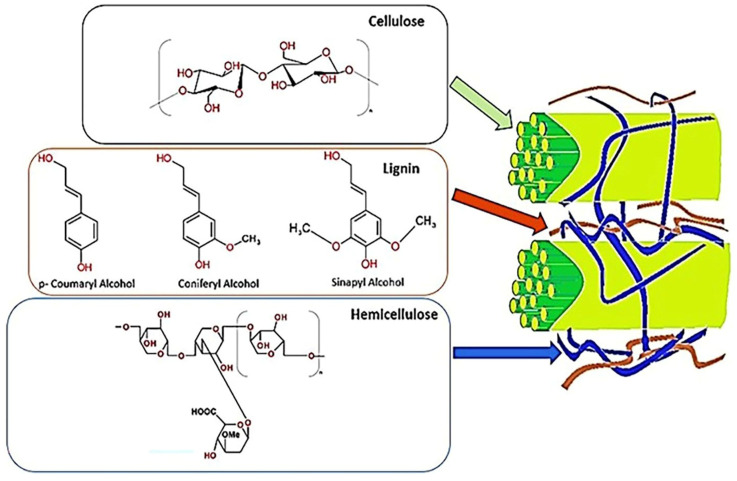
Composition of a lignocellulosic biomass material showing cellulose, lignin, and hemicellulose.

Globally, agricultural residue management is a significant challenge, with many crop residues being burned in the fields rather than utilized. Around 4 billion tonnes of agricultural residue are generated worldwide annually, and it is estimated that nearly a quarter of this—about 1 billion tonnes—is burned directly in the fields. This practice is particularly prevalent in India, China, and Brazil, contributing significantly to air pollution and greenhouse gas emissions. A quarter of the approximately 611 million tonnes of agricultural residue produced in India each year is burned in the fields rather than used [1]. Large-scale crop residue burning like this around October and December causes severe air pollution in Delhi and the surrounding regions. Crop burning was found to be a 12 times greater source of pollution in November 2018 than the upper limit for healthy air. Residents in this area are in danger of disease, as seen by the rise in respiratory illnesses [2]. The Indian government has launched several initiatives to transform agricultural waste materials into goods with additional value to lessen this risk. Farmers prefer to collect and sell straw rather than burn it if they receive a fair price. In addition, it will develop a sustainable ecology for village communities and increase the income of small families and marginalized farmers. An estimated 200 billion tonnes of lignocellulosic biomass are produced annually from agricultural wastes and forest residues [3]. The principal components of these biomass materials are extractives, cellulose, hemicellulose, lignin, and many inorganic elements, as shown in Figure 1. Agricultural waste is cheap, readily available, and renewable. These agricultural by-products may be sources of fuels, chemicals, fibres, and other industrial uses [4]. Rice straw, wheat straw, barley straw, corn stover, coconut coir, pineapple, sugarcane bagasse, and banana leaves are a few low-cost agricultural wastes that may be found in India [5].

The source of lignocellulosic biomass is agricultural and forestry waste. Rice straw, sugarcane bagasse, corn stover, corn stalks, and wheat straw are among the agricultural leftovers that have been the subject of substantial research, in addition to biopolymeric nanocomposites for the removal of dyes [6,7,8,9]. Studying the structure and characteristics of lignocellulosic biomass is difficult due to its multiscale complexity and diversity. The three main polymers in the biomass are lignin, hemicellulose, and cellulose. There is also a trace amount of ash and extractives. The uneven distribution of these three main components within the cell wall forms the skeleton, connecting material, and hard solids. The characteristics and possible uses of biomass are determined by the significant variations in the amounts of lignin, hemicellulose, and cellulose that are present. There is some variation in the cellulose concentration (40–60%), hemicellulose content (15–30%), and lignin content (10–25%). Agricultural residues have more ash and extractives than wood biomass, typically comprising a more significant proportion of the three primary components [10].

### Lignocellulosic Biomass Material: Overview and Characterization

Cellulose is the primary structural element that gives plant cell walls and fibre strength and stability. It is a syndiotactic linear polymer made up of β-(1,4)-D-glucose units that are polydisperse. Long molecular bundles resembling threads are formed when the cellulose chains combine. Hydrogen interactions between the oxygen and hydroxyl groups of nearby molecules serve to stabilize these molecules laterally [11]. Because of these bundles’ extremely regular molecular arrangement, cellulose displays a crystalline X-ray diffraction pattern. The resulting stable structure possesses outstanding mechanical properties, which include a high Young’s modulus (138 GPa) in the crystal region and a very low coefficient of thermal expansion (10^−7^ K^−1^) along the longitudinal direction [12].

Cellulose has the following chemical formula: (C_6_H_10_O_5_)n. It comprises β-1, 4 linkages connecting units of (1, 4)-D-glucopyranose and has a molecular weight of approximately 100,000. In nature’s crystalline and amorphous zones, cellulose molecules are bundles that combine to form micro-fibrils. The amount of cellulose in a fibre affects its characteristics and suitability for different uses. While cellulose is also a raw ingredient in the textile and fibrous chemical industries, fibres with a more significant cellulose percentage are often selected for pulp and paper [13]. Among lignocellulosic materials, hemicellulose is the second largest biomolecule, with an average molecular weight of 30,000. 1,4 glycosidic linkages hold the five- and six-carbon sugars that make up hemicellulose, a heteropolymer with a branching structure [14]. The hemicellulose fraction gives the biomass matrix its rigidity as a binding agent among the lignin and cellulose fractions. Cellulose and hemicellulose are more valuable sources of many chemicals with a high commercial value because they contain reducing sugars [13]. Since hemicellulose is readily hydrolysed into fermentable sugars, the biomass by-product with a greater hemicellulose concentration is preferred for producing ethanol and other fermentation products [15]. Lignin is the most minor and complicated biomass component at 10–25% of the total weight. The polymer makes plant cell walls stiff, preventing pathogenic microorganisms from attacking the cellulose polymer [16]. Lignin acts as a glue by occupying the surrounding space between the cellulose and hemicellulose complex [4]. It is a three-dimensional macromolecule composed of sinaphyl, coniferyl, and p-coumaryl alcohol. C-C and C-O-C connections bind these monolignols—the S, G, and H units—together [13].

The textile industry, a primary global sector producing over 100 million tons of fibre annually, significantly contributes to environmental pollution, mainly through wastewater generation. This industry, with leading producers including China, India, and the European Union, generates approximately 1.7 million tons of wastewater daily, primarily from dyeing processes. This wastewater is often heavily polluted, with chemical oxygen demand (COD) levels reaching up to 2000 mg/L, compared to 200–500 mg/L in typical municipal wastewater. The textile industry is a major water consumer, utilizing around 79 billion cubic meters annually for various processes. The wastewater typically contains high concentrations of synthetic dyes, heavy metals, and other chemicals, many of which are non-biodegradable and pose severe environmental and health risks if untreated. These figures underscore the immense scale of the pollution problem within the textile sector and highlight the urgent need for effective wastewater treatment solutions and sustainable practices to mitigate the industry’s environmental impact [17].

**Figure 2:**
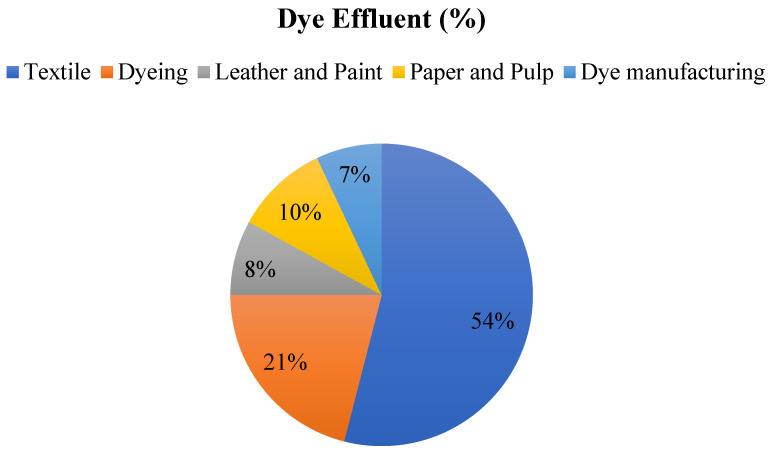
Dye effluent (%) of various industries.

Around 0.8 million tons of various dyes are produced annually, with an estimated 10–15% of this amount ending up as effluent in rivers, ponds, and lakes. The textile industry releases over half (54%) of the dye effluent worldwide. Other industries contributing to dye effluent include dyeing (21%), paper and pulp (10%), leather and paint (8%), dye manufacturing (7%), and sectors such as printing, food, rubber, petroleum, pigments, cosmetics, plastic, and pharmaceuticals, as illustrated in Figure 2 [17].

Textile effluents contain a range of dyes used in the industry, each with unique properties. Azo dyes, including direct, acid, and reactive types, are widely used, while anthraquinone dyes offer durability for outdoor fabrics. Sulphur dyes, known for their cost-effectiveness, colour cellulose fibres, and indigo dyes are used for denim. Reactive dyes form strong bonds with fibres, and dispersed dyes are used for synthetic materials like polyester. Acid dyes colour protein fibres and basic dyes target acrylic fibres. Alongside dyes, textile wastewater includes heavy metals, surfactants, and salts. Proper treatment is essential to mitigate environmental impact and protect ecosystems [18]. 

The dye concentration reported in textile effluent ranges from 10 to 250 mg/L [19]. However, this range can vary for different textile industries. The concentration of reactive dyes discharged in textile effluent can range between 60 mg/L and 200 mg/L [20], and for Acid Orange 10 dye, it is 45 mg/L [18]. The presence of dyes in effluent can harm aquatic species, plants, and human beings. When the dyes mix with water sources (ponds, lakes, and rivers), they form a thick visible layer on the surface due to their low density (0.8 kg/m3). This layer blocks the penetration of sunlight, which is necessary for aquatic animals or underwater species’ respiration or photosynthesis [21]. The dyes present in effluent also affect soil productivity due to the clogging of pores in the soil. The water quality gradually declines and thus it becomes unsuitable for daily consumption [17]. 

Moreover, the dye effluent can cause skin problems in human beings. If the dye comes in contact with the human eye, it can injure the eye. The inhalation of these harmful chemicals can create breathing problems, sweating, nausea, or vomiting. The dye chemicals are carcinogens and have long-term effects on the human body [22]. Therefore, removing the dyes from the effluent is necessary before it is discharged into water bodies. Laws have been passed regarding the concentration of toxic substances released into water bodies [17]. The international permissible limits for different pollutants are as follows: colour > 1 ppm, pH levels from six to nine, a temperature < 42 °C, BOD > 30 mg/L, COD > 50 mg/L, and suspended solids > 20 mg/L. Reusing the treated water when dyeing textiles is also recommended instead of disposing it into the environment. Various treatment methods, including biological, chemical, and physical approaches, have eliminated textile dyes from wastewater. The choice of treatment method depends on the composition and type of wastewater. Several physicochemical and biological processes have effectively removed dyes from industrial effluents. These methods include photocatalytic degradation, membrane filtration, biological treatment, irradiation, ultrasonic-assisted adsorption, and conventional adsorption [23,24,25,26,27,28].

Additional methods for decolourizing textile effluents include precipitation, ion exchange, and reverse osmosis [29]. Despite their effectiveness, these methods face limitations such as high operational costs, lengthy treatment times, and complex processes. As a result, there has been growing interest in finding alternative techniques to address these drawbacks. Adsorption is a promising technique for removing contaminants from water due to its cost-effectiveness, wide availability, user-friendly nature, and scalability [30,31]. Various materials have been used as adsorbents, including clay minerals, biomass, synthetic polymers, biopolymeric nanocomposites, activated carbon, and composites [21]. Activated carbon is the most used adsorbent due to its highly microporous structure and high surface-area-to-mass ratio. However, its slow adsorption rate, high cost, and inability to regenerate have restricted its use for large-scale wastewater treatment [32,33].

Researchers have explored using low-cost lignocellulosic biomass as natural adsorbents for removing synthetic dyes from wastewater [34,35,36]. These biomasses are plentiful, inexpensive, and sourced from renewable materials. Both raw and processed agricultural and biomass waste materials have been utilized. Examples of raw-form adsorbents include dragon fruit peel, pineapple stem, oak acorn peel, white pine sawdust, potato peels, luffa cylindrica, sugarcane bagasse, coconut coir dust, cashew nutshell, mango leaf powder, *Haloxylon recurvum* plant stems, eggshell, and cactus fruit bark [37,38,39,40,41,42,43,44,45,46,47,48,49]. In some instances, adsorbents were pretreated with acids or alkalis before use. Examples of these treated adsorbents include rejected tea altered with NaOH [50], cotton straw activated with sulphuric acid [51], activated carbon from walnut wood treated with nitric acid [52], banana peels activated with NaOH [53], and pomelo skin and jackfruit peel activated with carbon using microwave-induced NaOH treatment [54]. Similarly, activated carbon made from biomass, such as bamboo and coconut shells, has shown notable adsorption effectiveness for crystal violet dye, according to Kumar and Jena’s findings [55]. Studies conducted by Hameed et al. and Annadurai et al. have investigated the usage of materials such as rice husk, banana peel, and orange peel, and they have shown noteworthy removal efficiencies for many dyes, including Methyl orange, Malachite green, and Congo red. The efficacy of these natural adsorbents is influenced by factors such as pH, temperature, initial dye concentration, and contact time, which have been systematically investigated to optimize the adsorption process. Many variables that have been thoroughly studied to optimize the adsorption process, including pH, temperature, initial dye concentration, and contact time, affect how effective these natural adsorbents are [56,57].

The recent literature on using biomass-based materials for dye removal from wastewater highlights their significant potential and variability in performance. For example, lignocellulose-based materials such as sugarcane bagasse and rice husk demonstrate notable dye adsorption capacities, with values reaching 1.83 mg/g and 40.6 mg/g, respectively, for methylene blue (MB) and other dyes. Agro-biomass byproducts have also shown promising results; psyllium seed powder achieved an impressive adsorption capacity of 206 mg/g for Reactive Orange 16, while mango leaf powder exhibited a high capacity of 708 mg/g for Acid Yellow 99. Additionally, eggshell waste has been utilized effectively, with adsorption capacities increasing from 21% to 58% for Direct Blue 78 when using NaOH as a desorption agent. Conversely, fly ash, which is widely used, has shown lower capacities with adsorption values of 4.3 mg/g for Reactive Black 5 and 2.1 mg/g for Reactive Red 23. These findings underscore the diverse efficacy of various biomass-based adsorbents and highlight the potential of agricultural and industrial byproducts as sustainable solutions for dye removal from wastewater [58,59].

The literature on using lignocellulosic biomass for dye removal from textile wastewater is limited to several key areas. There is insufficient data on the efficiency and scalability of these materials in various real-world applications, and there is a lack of comprehensive studies comparing different types of lignocellulosic biomass. Moreover, the mechanisms involved in dye adsorption are not well understood, and there is a need for research on the long-term environmental impact and economic feasibility of these methods.

## 2. Dye Removal Methods

### 2.1. Biological Processes

Biological processes for dye removal involve aerobic and anaerobic treatments, biosorption, and bioaugmentation. Aerobic treatments use oxygen-dependent microorganisms, while anaerobic treatments work without oxygen to degrade complex dyes. Due to their high surface area and functional groups, biosorption employs biological materials like algae to adsorb dyes. Bioaugmentation enhances dye degradation by adding specific microorganisms to wastewater. These methods provide diverse strategies for effective dye removal based on wastewater characteristics. These involve using various resources such as bacteria, enzymes, yeast, algae, microbes, and plants to treat wastewater [60]. Among all sources, algae are the most widely used for biosorption as they contain lipids, proteins, and other functional groups like amino and carboxylic acids. Moreover, algae have a vast surface area, showing a high binding affinity for various contaminants [61].

A study on the biomass obtained from algae D. Antarctica reported an adsorption capacity of 702.9 mg/g for (MB) dye under optimum conditions [62]. Similarly, biomass (Laminaria japonica) obtained from renewable brown algae exhibited an adsorption capacity of 549.45 mg/g for MB under-maintained optimum conditions.

The efficiency of biological treatment for dye removal is influenced by factors such as pH, temperature, dye concentration, nutrient availability, microbial community composition, oxygen levels, retention time, and the presence of inhibitory substances. Optimal pH and temperature ranges enhance microbial activity while adequate nutrients support growth. High dye concentrations and inhibitory chemicals can hinder treatment efficiency. Proper oxygen levels are crucial for aerobic processes, and a sufficient retention time improves dye removal. Understanding these factors is essential for optimizing biological dye removal methods [63].

While these procedures are sustainable and kind to the environment, they are generally slower than chemical approaches and necessitate precise control over parameters like pH, temperature, and nutrient availability. Furthermore, not all dye molecules may be eliminated by biological processes, leaving residual pollution. Despite these obstacles, biological technologies’ sustainability and non-toxic character make them a viable field for additional research and development [62].

### 2.2. Chemical Methods

Advanced oxidation processes, photochemical reactions, Fenton reactions, ozonation, electrochemical destruction, oxidation, and ultraviolet irradiation are some techniques used and are influenced by factors like pH, temperature, dye concentration, catalyst presence, oxidant dose, reaction time, interference by other substances, and energy input. Chemical dye removal techniques typically cost more than alternative techniques. Furthermore, because these techniques require specialized equipment and considerable amounts of electrical energy, they are challenging to execute [64]. Large-scale chemical usage is another important problem. When chemical dye removal is performed, it can lead to the development of dangerous secondary pollutants and other negative features [17].

Recent studies have focused on enhancing efficiency and sustainability. For instance, Li et al. explored novel catalysts for advanced oxidation [65], and Zhang et al. investigated sunlight-driven photocatalysis for cost-effective dye removal [66]. Other studies have optimized Fenton processes [67], integrated ozonation with biological treatments [68], and developed advanced electrodes for electrochemical destruction [69].

Concerns about sustainability have also been raised by the energy-intensive nature of chemical processes, which need a significant amount of electrical power. Chemical use can also produce dangerous secondary pollutants, increasing environmental risks. The large-scale implementation of these techniques can be complex because of the technical know-how and secondary pollution risk. It still needs to be easier to balance chemical processes’ efficiency and their effects on the environment and the economy [70].

### 2.3. Physical Methods

Compared to chemical and biological dye removal techniques, these are the most widely used techniques for removing dye because of their ease of use, effectiveness, and low chemical requirements. Factors affecting these methods include the type and surface area of the adsorbent, membrane pore size, flow rate, dye concentration, pH, temperature, and the presence of competing ions and particles [17]. Various physical methods are adsorption, membrane filtration, ion exchange, reverse osmosis, and coagulation and flocculation. Among all the adsorption methods, the physical adsorption method is common, effective, and easy to implement, and it can easily remove a variety of dyes or a mixture of dyes.

Recent studies have focused on enhancing these techniques. For example, Wang et al. developed high-surface-area biochar for dye adsorption [71], while Li et al. improved nanocomposite membranes for higher dye rejection rates. These advancements highlight the potential for more efficient and sustainable dye removal processes [72].

Physical techniques often include less chemical input, which lowers the possibility of secondary pollution. However, equipment such as membrane filters might have substantial setup costs initially, and these filters must be replaced and maintained regularly. Adsorbents may also require regeneration or replacement over time due to their limited lifespan. Despite these limitations, physical techniques provide a workable and efficient way to remove the dye, and research is still being conducted to increase the adsorbent materials’ affordability and durability [72].

## 3. Dye Removal via Adsorption

Among the dye removal techniques, adsorption is favoured by various dyes due to its low cost, affordability, and minimal maintenance [73]. The adsorbent is reusable for several treatment procedures. The high cost of adsorbents is the only drawback of this approach [74,75]. Particles collect at the interface of two phases, gas–liquid, gas–solid, liquid–liquid, and liquid–solid, by the non-reactive mass transfer process known as adsorption [76]. Adsorptions are physical (physisorption) or chemical (chemisorption). Weak electrostatic forces, like dipole–dipole and van der Waal interactions, in which the bonds are easily broken, cause physical adsorption, which has a low enthalpy of adsorption (ΔH = 20 to 40 kJ/mol). Many adsorbate layers are deposited onto the adsorbent during this quick and reversible process. Physisorption occurs at a temperature lower than the adsorbate’s boiling point [77].

On the other hand, chemisorption happens when the adsorbate and adsorbent establish covalent bonds because of the transfer of electrons [77]. With a high adsorption enthalpy (ΔH = 80–200 kJ/mol), it is a sluggish, irreversible, single-layer process [78]. As the temperature rises in this process, adsorption first rises and subsequently falls. Physical adsorption frequently accounts for the majority of adsorption [79]. The binding of atoms, ions, or molecules on a solid surface’s active sites is known as adsorption. Adsorption generally requires little energy input, and the kinetic equilibrium and adsorbent composition determine how well dyes are removed [80].

### 3.1. Factors Affecting Adsorption

**Figure 3:**
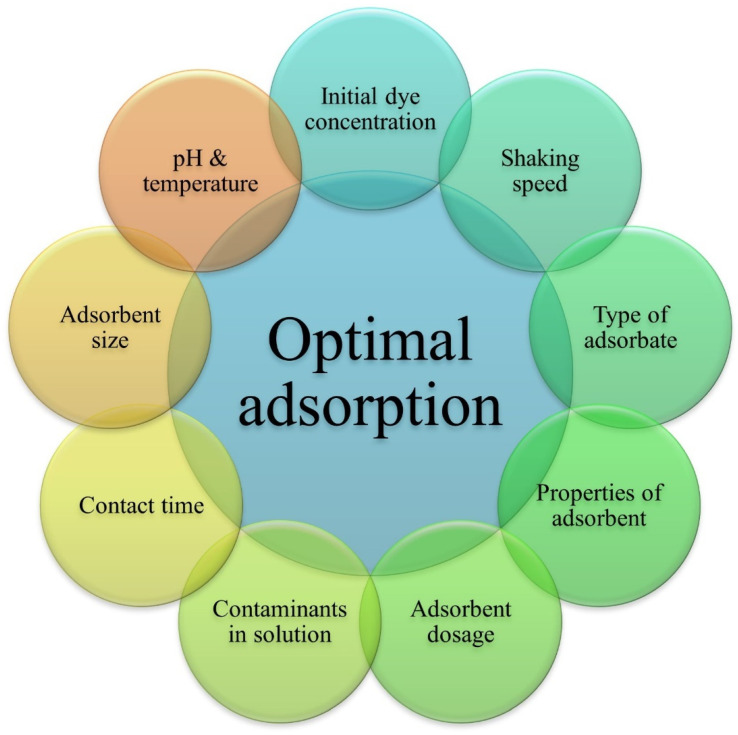
Factors affecting the adsorption process.

The adsorption performance is affected by physicochemical factors. Besides the adsorbent and treatment bath parameters, adsorbate properties, like molecular weight, molecular structure, and size, also affect the adsorption performance [80]. The optimization of all these parameters plays a role in the efficient removal of dye from effluents, as shown in Figure 3.

#### 3.1.1. Dye Concentration

One important aspect influencing the efficacy of dye removal is the initial concentration of dye in the effluent. The initial dye concentration has a direct bearing on the efficacy of dye removal (R%) and the maximum amount of dye adsorbed at equilibrium (qe) [81]. Table 1 summarizes research findings on the impact of the starting dye concentration on adsorption. Owing to the saturation of active sites on the adsorbent surface, the percentage of dye removed steadily drops as the original dye concentration rises. The adsorption is propelled by the initial concentration of the dye, which facilitates the process of mass transfer and diffusion from the solution to the adsorbent’s free surface [82]. When the initial concentration is high, it may result in a lower adsorption efficiency. At lower concentrations, the ratio of adsorptive active sites to dye molecules is higher, allowing the dye molecules to interact with the adsorbent and facilitate the dye’s removal from the solution [83]. To remove Congo Red dye using cocoa bean shells, a study found an inverse link between the initial dye concentration and the adsorption efficacy. This negative effect shows that the adsorbent’s ability for adsorption is reduced as the dye concentration rises. This was explained by the fact that the resistance mechanism to dye removal, which acts as the adsorption process controller, increases the adsorption capacity at equilibrium with an increase in dye concentration [84].

Table 1 presents the influence of the initial dye concentration on the adsorption capacity and dye removal efficiency for various adsorbents and dyes. For methylene blue (MB), Algerian Palygorskite shows an adsorption capacity ranging from 2.5 to 10 mg/g with a dye removal efficiency reaching up to 97% at concentrations between 3 and 30 mg/L after 5 min of reaction time. *Haloxylon recurvum* plants, used for Acid Brown dye, exhibit an adsorption capacity between 2.8 and 10 mg/g at concentrations of 10 to 60 mg/L over 180 min, though their dye removal efficiency is not provided. Fava bean peels, also used for MB, achieve an unspecified adsorption capacity but demonstrate dye removal efficiencies between 70% and 95% for concentrations ranging from 3.6 to 100 mg/L over 70 min. Corn silk, applied to Reactive Blue 19 and Reactive Red 128, shows variable adsorption capacities from 2 to 71 mg/g and 2 to 63 mg/g, respectively, with its dye removal efficiencies not reported. Spent tea leaves, used for Reactive Black 5, achieve an adsorption capacity between 24.8 and 6.7 mg/g, with a dye removal efficiency ranging from 43% to 99% at concentrations of 50 to 100 mg/L and reaction times between 5 and 200 min. *Citrus Limetta* peel for Malachite Green displays an adsorption capacity from 0.17 to 4.7 mg/g and a high removal efficiency between 95% and 97% at concentrations of 5 to 25 mg/L over 10 to 60 min. Mango stone biocomposite for Crystal Violet shows an adsorption capacity between 25 and 352 mg/g, though its dye removal efficiency is not specified. Moringa oleifera seed for Reactive Red 120 achieves an adsorption capacity ranging from 18.5 to 174 mg/g (dye removal efficiency not provided) at concentrations of 10 to 100 mg/L over 30 min. Olive leaf powder for Crystal Violet exhibits an adsorption capacity between 5 and 45 mg/g, with its dye removal efficiency not specified, at concentrations of 10 to 100 mg/L and reaction times from 5 to 70 min. This table highlights the significant variation in adsorption performance based on initial dye concentration and adsorbent type, emphasizing the need to tailor adsorbent selection and operational conditions to optimize dye removal processes.

#### 3.1.2. pH

The pH of the solution is another important factor in the dye removal process. The activity of functional groups, competition with coexisting ions in solution, surface charge of the adsorbent, adsorption process, and dissociation of dye molecules are all impacted by the pH of the solution. Additionally, the pH of the solution alters the dye’s chemical structure. It influences the amount of electrostatic charge that dye molecules transfer, which in turn influences the rate of adsorption [93]. HCl and NaOH are used to modify the dye solution’s pH. Adding HCl to the solution causes the adsorbent’s surface to become protonated, better facilitating the anionic dye’s ability to bind to it by electrostatic bonding. Adding NaOH leads to deprotonating of the biomass surface, creating a primary medium. As a result, the negatively charged adsorbent and anionic dyes repel one another. Therefore, the adsorption of cationic dyes is advantageous in the basic media. MB and Malachite Green colours were eliminated using eggshell powder as an adsorbent. The dye removal efficiency (%) increased from 14.8 to 75.1 and 89.9 to 97.9, respectively, when the pH climbed from two to ten [94]. For MB, a comparable pattern was also observed in natural olive stone. The adsorption capacity rose from 18 to 20 (mg/g) as the pH rose from 2 to 12 [95]. On the other hand, anionic dyes exhibited the reverse trend, and it was discovered that an acidic medium is beneficial for them. For instance, Bromophenol Blue and Congo Red dyes were absorbed using eggshell powder adsorbent. The dye removal efficiency (%) falls from 98.7 to 93.1 and 67.6 to 1.2 when the pH rises from two to ten [94]. Analogous outcomes were also observed when anionic dyes were removed from spent mushroom debris, and it was discovered that a pH of two is the optimal pH for dye removal. According to the study, the dye removal rates for Direct Red 5B, Direct Black 22, Direct Black 71, and Reactive Black 5 were 95%, 98%, 95%, and 96%, respectively [96].

#### 3.1.3. Adsorbent Dosage

The adsorbent dosage determines the adsorbent capacity for a given dye concentration [97]. Studies on how the adsorbent dose affects the dye removal efficiency are compiled in Table 2. The dye removal efficiency rises with increasing adsorbent dosage. More adsorption active sites are available when the adsorbent dose is increased at a fixed dye concentration because this increases the amount of active surface area available for adsorption [98]. On the other hand, the adsorption capacity (mg/g) falls with increasing absorbent dosage. This could be explained by two factors: adsorbent particles may aggregate, decreasing the available surface area, and some active sites stay unsaturated despite an increase in the number of sites accessible for adsorption.

Table 2 explores the effect of adsorbent dosage on the adsorption capacity and dye removal efficiency for various adsorbents and dye types. For methylene blue (MB), walnut shell ranging from 0.5 to 2 g/L results in an adsorption capacity between 179 and 48 mg/g, with the dye removal percentage not specified. The alginate/rice husk bio-composite, with dosages from 0.1 to 1 g, achieves an adsorption capacity of 338 to 145 mg/g and a dye removal efficiency ranging from 15% to 89%. Raw petroleum coke, used for Congo Red, exhibits a dosage range of 4 to 24 g/L, with a dye removal efficiency between 10% and 60%, although the adsorption capacity is not specified. Municipal solid waste compost ash effectively removes Reactive Red 198 with a dosage of 0.5 to 2 g/L, showing a dye removal efficiency between 79% and 93%. The mucilage of Salvia seed, used for Cationic Blue 41, achieves an adsorption capacity from 34.2 to 6.74 mg/g and a dye removal efficiency ranging from 34.2% to 54% with a dosage of 0.5 to 4 g/L. For Remazol Brilliant Violet 5R, eggshell shows a dosage-dependent adsorption capacity from 2.9 to 0.75 mg/g and a dye removal efficiency between 74.6% and 93.8% with dosages of 0.5 to 2.5 g. Similarly, calcined eggshell, with dosages from 0.5 to 2 g, achieves an adsorption capacity from 3.5 to 0.96 mg/g and a higher dye removal efficiency between 89.8% and 96.3%. This table highlights the importance of optimizing adsorbent dosage to enhance both adsorption capacity and dye removal efficiency in wastewater treatment.

#### 3.1.4. Adsorbent Size

The entire surface area of the adsorbent per unit mass is influenced by its size, which makes it an important aspect of the adsorption process. Table 3 presents a summary of research on this topic. An increased specific surface area is the outcome of smaller adsorbent sizes. In addition, the porosity, structure, and adsorbent size all play a role [106]. Due to the increased number of active sites for binding the dyes, the dye removal percentage increases with the adsorbent size. Three factors determine how the adsorbent size and adsorption capacity (mg/g) relate to one another [107]: the adsorbent’s porosity and crystallinity, the adsorbate’s ionic charge and chemical structure, and the adsorbent’s capacity to generate hydrolysed species.

Table 3 examines the impact of adsorbent particle size on dye adsorption and removal efficiency. It compares various adsorbents, such as cabbage waste powder, macadamia seed husks, coconut shell activated carbon, coffee husks, and a biopolymer-based nanocomposite, across different dye types including Congo Red, Reactive Black 5, Direct Yellow 12, Crystal Violet, and methylene blue (MB). The data show that the dye removal efficiency varies with the adsorbent size; for instance, macadamia seed husks demonstrate a wide range of removal efficiencies from 99% to 33% depending on the particle size, while coffee husks achieve a removal efficiency between 96% and 90%. Coconut shell activated carbon exhibits a decreasing adsorption capacity with increasing particle size, ranging from 5.5 mg/g to 3.5 mg/g for Direct Yellow 12. The biopolymer-based nanocomposite shows a high dye removal efficiency between 99% and 86% for methylene blue, reflecting the significant role of particle size in optimizing adsorbent performance. These findings underscore the need to carefully select and optimize adsorbent sizes to enhance the effectiveness of dye removal in wastewater treatment.

#### 3.1.5. Temperature

One crucial element that influences the effectiveness of adsorption is the temperature of the solution. Changing the reaction’s nature—from endothermic to exothermic or vice versa—modifies the process [112]. Depending on the absorbent and adsorbate, a solution’s temperature can affect the adsorption efficiency. The rate of a chemical reaction often rises as the temperature rises. This is advantageous for chemisorption since it increases the adsorption efficiency at higher temperatures. Physical adsorption decreases with temperature. When the temperature changes, two processes take place: endothermic and exothermic. 

*Exothermic*: As the temperature rises in this process, the adsorption effectiveness falls. An increased temperature reduces the removal efficiency by weakening the adsorptive link between the dye and active adsorbent sites [113]. Because of the system’s increased ion mobility due to heat, this kind of process influences the diffusion process.

*Endothermic*: Since more active sites become available due to the activation of adsorbent surfaces, the effectiveness of dye removal in this process rises as the temperature rises. Raising the temperature improves the adsorption capacity (mg/g), which could be explained by the increased mobility of significant dye ions [114]. The influence of the bath temperature on dye adsorption effectiveness was found in the investigation of the removal of MB dye utilizing palm tree debris as an adsorbent. The adsorption capacity increased from 21 to 35 (mg/g) as the temperature rose from 20 °C to 70 °C, demonstrating that the process was endothermic [115]. Similar findings were observed when using Mansonia wood sawdust as the adsorbent to remove methyl violet, a basic colour. When the temperature rose from 26 °C to 56 °C, the adsorption capacity increased from 18 to 23 mg/g [116], indicating that the adsorption process was endothermic. Nevertheless, another study that used the cone biomass of *Thuja orientalis* as an adsorbent to remove Acid Blue 40 revealed that higher temperatures reduce the adsorption capacity, suggesting that the adsorption process was exothermic. The adsorption capacity (mg/g) dropped from 8.22 to 5.51 when the temperature rose from 20 °C to 40 °C [117]. 

#### 3.1.6. Miscellaneous Factors 

Several other key physicochemical factors influence the efficiency of the adsorption process for dye removal from wastewater:

*Shaking Speed:* The agitation speed during adsorption impacts the contact between the adsorbent and adsorbate. Higher shaking speeds generally enhance mass transfer rates and improve the adsorption efficiency by increasing the interaction between dye molecules and the adsorbent surface. For example, Zhang et al. found that optimizing the shaking speed significantly improved the removal efficiency of Reactive Red 198 using activated carbon [118].

*Type of Adsorbate:* The characteristics of the dye being removed, such as its molecular weight, structure, and charge, affect the adsorption process. Different dyes interact differently with adsorbent materials due to their varying chemical properties. For instance, Mahmoud et al. highlighted that reactive dyes with larger molecular structures exhibited slower adsorption rates than smaller, simpler dyes [119].

*Properties of Adsorbent:* The physical and chemical properties of the adsorbent, including surface area, pore size, and chemical composition, are critical for effective dye removal. Adsorbents with higher surface areas and appropriate pore sizes typically show a better performance. For example, the study by Yadav et al. demonstrated that biochar with an increased surface area and porosity significantly enhanced the removal of Azo dyes from wastewater [120].

*Contaminants in Solution:* Other contaminants or competing ions in the solution can affect the adsorption efficiency. These substances may compete with dye molecules for adsorption sites or alter the adsorption capacity of the material. Liu et al. investigated how the presence of metal ions impacted the adsorption of various dyes, showing reduced efficiency due to competition for active sites [121].

*Contact Time:* The duration of contact between the adsorbent and the dye solution influences the extent of the dye removal. Longer contact times generally allow for more significant adsorption, reaching equilibrium more effectively. According to the research by Chen et al., increasing the contact time improved the adsorption capacity for both basic and acidic dyes, though with diminishing returns after a certain period [122].

### 3.2. Adsorption Isotherms

The adsorption mechanism of dyes on textile substrates is studied using adsorption isotherms. When the bath is at equilibrium, the isotherm shows how the dye molecules are distributed across the liquid and solid phases. The experimental data are fitted to various isotherm models to determine which isotherm model is best for design purposes [123]. Langmuir, Freundlich, Temkin, Dubin–Radushkevich, Hasley, and Harkins–Jura are the different isotherms. Langmuir and Freundlich are the two isotherms for dye adsorption most frequently examined in the literature [124].

- Langmuir Isotherm

The Langmuir model, which explains the monolayer adsorption process, states that adsorption takes place on a homogeneous/homogenous surface with sites on the adsorbent of the same kind. Equation (1) provides the model’s nonlinear form. Equation (2), the Langmuir model’s dimensionless constant separation factor (R_L_), tells us whether the adsorption process is feasible. R_L_ = 1 (linear), R_L_ = 0 (irreversible), R_L_ > 1 (unfavourable), 0 < R_L_ < 1 (feasible and favourable), and so on. The character of the adsorption process can be determined as follows [125]:

(1) $$q_{e}=\frac{q_{max}K_{L}C_{e}}{1+K_{L\;}C_{e}}$$

(2) $$R_{L}=\frac{1}{1+K_{L\;}C_{i}}$$

- Freundlich Isotherm

The Freundlich isotherm can be used in dye fibre systems in which multilayers or heterogeneous areas experience adsorption. Equation (3) is the model’s nonlinear form.

(3) $$q_{e}=K_{F}{C_{e}}^{1/n}$$

The variables C_e_ and q_max_ represent the equilibrium dye adsorption capacity on the adsorbent (mg/g and maximum monolayer adsorption capacity, respectively), while K_L_ is the Langmuir isotherm constant (L/mg). The Langmuir curve’s slope and intercept (ce vs. c_e_/q_e_) were used to calculate these constants: K_L_ = slope/intercept, and q_max_ = 1/slope. The constants denoting the Freundlich features are 1/n and K_F_ [(mg/g) (L/mg)1/n]. K_F_ = 10 antilog (intercept) and 1/n = the slope of the Freundlich curve (log c_e_ vs. log q_e_) were used to compute these constants [126].

### 3.3. Adsorption Kinetics

One crucial component that characterizes the efficiency of adsorption is kinetics. It is the solute’s rate of adsorption as well as the adsorbate’s residence duration on a solid–liquid surface [127]. The pseudo-first-order and pseudo-second-order kinetic models are the two most often utilized types. According to the pseudo-first-order kinetic model, the variation in saturation concentration over time determines the solute uptake rate. Kinetics are frequently seen to obey the Lagergren pseudo-first-order rate equation when adsorption happens by diffusion. Equation (4) displays the pseudo-first-order rate equation [128].

(4) $$\ln(q_{e}-q_{t})=\ln q_{e}-\frac{k_{1}}{2.303}t$$

where k_1_ (min^−1^) is the adsorption rate constant, and q_e_ and q_t_ (mg/g) denote the amount of dye adsorbed at equilibrium and at time t (min), respectively. Based on the linear plots of ln (q_e_ − q_t_) vs. t, the values of k_1_ and q_e_ were determined (q_e_ = exp Intercept, slope = −K_1_) [129]. 

The chemisorption, or chemical sorption, is assumed to be the rate-limiting step in the pseudo-second-order kinetic model. Throughout the entire adsorption range, it forecasts the adsorption behaviour. Here, the adsorption capacity rather than the adsorbate concentration determines the adsorption rate. Equation (5) represents the pseudo-second-order kinetic model’s equation.

(5) $$\frac{t}{q_{t}}=\frac{1}{k_{2}{qe}^{2}}+\frac{1}{q_{e}}t$$

The pseudo-second-order constant is denoted by k_2_ (g (mg min)^−1^), while Q_e_ represents the equilibrium adsorption capacity (mg/g). The slope and intercept of the linear plot t/q_t_ vs. t can be used to determine these experimentally (K_2_ = Slope^2^/Intercept, q_e_ = 1/slope) [129].

### 3.4. Thermodynamics Study

Thermodynamic parameters, including entropy, Gibbs free energy, and enthalpy, are computed in adsorption studies to describe response behaviour and provide insight into the adsorption mechanism. Equation (6) can be utilized to ascertain the parameters derived from experimental data to assess the viability of the adsorption process [130].

(6) $$\Delta G{}^{\circ}=-RT\log K_{L}$$

This can be linearized to Equation (7).

(7) $$\log K_{L}=\frac{-\Delta G{}^{\circ}}{RT}=\frac{\Delta S{}^{\circ}}{R}-\frac{\Delta H{}^{\circ}}{RT}$$

(8) $$K_{L}=\frac{q_{e}}{C_{e}}$$

where T (K) is the absolute temperature, R is the universal gas constant (8.314 J/mol K), and K_L_ is the equilibrium constant found using Equation (8) for each temperature. A straight line is produced by plotting log K_L_ vs. 1/T, and the slope and intercept of the plots can be used to calculate ∆H° and ∆S°, respectively. The adsorption process is either endothermic or exothermic, depending on whether the enthalpy value is positive or negative. Like this, the negative value of Gibbs free energy in the adsorption process shows the spontaneity or non-spontaneity of the adsorption process. The positive entropy value shows high unpredictability during adsorption [130]. 

### 3.5. Lignocellulosic Biomass Materials as Adsorbents

Research on the use, efficiency, and characteristics of various adsorbents has revealed that one of the main problems is the expense of the adsorbent utilized in the adsorption process [17]. Numerous alternatives, including affordable, eco-friendly, green, and biodegradable adsorbents, have been researched. The capacity of inexpensive adsorbents to extract different kinds of contaminants from aqueous solutions determines their efficiency. Essentially, the purpose of these adsorbents is to use waste materials from different sectors and agriculture to replace costly activated carbons. Such by-products’ sheer volume and toxicity usually cause them to have a lot of disposal issues. The utilization of waste as an inexpensive adsorbent has the potential to address environmental pollution in two ways. Firstly, trash could be produced in smaller quantities, and secondly, wastewater pollution could be reasonably decreased at a fair cost using inexpensive adsorbents made from these wastes [131]. Table 4 lists some raw (untreated) adsorbents based on lignocellulosic biomass materials, with the type of dye utilized and their maximal monolayer adsorption capacity. The adsorbents have had treatments applied to them or have not been treated.

Table 4 presents a comprehensive overview of raw adsorbents derived from lignocellulosic biomass materials and their effectiveness in removing dye from wastewater. It lists adsorbents like sugarcane bagasse, rice husk, brewers spent grain, orange albedo, and more, along with the types of dyes they target and their monolayer adsorption capacities, measured in milligrams per gram (mg/g). For instance, sugarcane bagasse shows an adsorption capacity of 1.83 mg/g for methylene blue, while mango leaf powder exhibits an exceptionally high capacity of 708 mg/g for Acid Yellow 99. Other notable adsorbents include banana peel powder and psyllium seed powder, which have capacities of 49.2 mg/g and 206 mg/g for Reactive Blue 5 and Reactive Orange 16, respectively. The table underscores the significant potential of these biomass materials in dye adsorption, highlighting the variation in adsorption efficiency depending on the type of adsorbent and dye. The referenced studies validate the data and provide a foundation for further research and application in wastewater treatment.

### 3.6. Desorption Studies

Desorption studies play a crucial role in understanding the adsorbent’s and adsorbate’s recovery and elucidating the underlying adsorption mechanism. These studies involve treating the spent adsorbent to regenerate it, thus ensuring its potential reuse. Table 5 is an overview of the various biomass adsorbents investigated in desorption or regeneration studies.

Regeneration poses a complex challenge as the effectiveness of desorption is contingent upon factors such as the type of adsorbent, adsorbate, and the specific adsorption process involved. Through the adsorption and subsequent desorption processes, researchers aim to assess the reusability of the adsorbent, a critical aspect of sustainable water treatment practices. Numerous studies have explored the efficiency of adsorbents in removing dyes from aqueous solutions through adsorption–desorption investigations. Various eluents are employed to facilitate the desorption of pollutants from spent adsorbents. These eluents encompass a diverse range of substances, including water, salts like NaCl, acidic solutions such as HCl and H_2_SO_4_, alkalis like NaOH and KOH, organic solvents like ethanol and acetone, chelating agents like EDTA, and organic acids such as tartaric acid and citric acid. Each eluent offers unique desorption properties, thus influencing the overall regeneration process and the subsequent reuse potential of the adsorbent [80].

Table 5 outlines the regeneration potential of various biomass adsorbents used for dye removal, focusing on their desorption efficiency and the agents employed. For instance, modified rice husk showed desorption efficiencies of 62.8 mg/g, 80.5 mg/g, 53.7 mg/g, and 75.3 mg/g for Direct Orange 26, Direct Red 31, Direct Blue 67, and Ever Direct Orange, respectively, with a 17% decrease in adsorption capacity after 10 cycles using H_2_O, NaOH, and Na_2_CO_3_ as desorption agents. Activated pistachio shell, targeting Acid Violet 17, saw its desorption efficiency drop from 94.6% in the first cycle to 75.8% in the third cycle when treated with NaCl, HCl, NaOH, and CH_3_COOH. Black and green olive stones used for methylene blue (MB) achieved desorption efficiencies of 92.5% and 88.1%, respectively, with acetic acid and ethanol. Modified orange tree sawdust showed a 16.5% efficiency with DI water and 58.6% with NaCl for MB dye. Egg shell’s efficiency for Direct Blue 78 increased from 21% in the first cycle to approximately 58% in the third cycle using NaOH. Mango leaf powder exhibited a 97% efficiency with NaOH and a 58.5% efficiency with NaCl for Acid Yellow 99. Lastly, *Dioscorea opposita Thunb*. (DOT) demonstrated a 93% desorption efficiency for Indigo Carmine using NaOH. These findings underscore the importance of selecting appropriate desorption agents to optimize the regeneration and reuse of biomass adsorbents in dye adsorption processes.

## 4. Challenges and Limitations

While lignocellulosic biomass materials show great promise as adsorbents for removing textile dyes from wastewater, several limitations and challenges need to be addressed to optimize their practical application.

*Variability and heterogeneity in structure:* The inherent heterogeneity of lignocellulosic biomass can lead to variability in adsorption efficiency. Different biomass sources, even from the same species, may have variations in cellulose, hemicellulose, and lignin content, impacting the uniformity and predictability of adsorption performance.

*Limited adsorption capacity:* The adsorption capacity of lignocellulosic materials can be lower compared to synthetic adsorbents, particularly for high concentrations of dyes. This limitation may necessitate larger quantities of biomass or pre-treatment modifications, potentially increasing costs and complexity.

*Chemical stability and durability:* Lignocellulosic materials may undergo degradation or chemical modification when exposed to certain pH levels, temperatures, or chemical treatments, affecting their adsorption efficiency and reusability. Ensuring the durability of these materials over multiple cycles of adsorption and desorption remains a significant challenge.

*Desorption and regeneration:* The desorption process is often less efficient, potentially limiting the reusability of the biomass materials. Inefficient desorption can lead to residual dye retention, reducing the materials’ effectiveness over time. Developing cost-effective and environmentally friendly regeneration techniques is crucial for their sustainable use.

*Scaling and practical application:* Scaling up from laboratory experiments to real-world applications poses logistical and economic challenges. The bulk handling, processing, and disposal of large volumes of biomass can be cumbersome. Additionally, there is a need for standardization in the preparation and usage protocols to ensure consistent performance across different treatment facilities.

*Economic and environmental considerations*: While lignocellulosic materials are often sourced from agricultural waste, the economic viability of collecting, transporting, and processing these materials must be carefully evaluated. Furthermore, the environmental impact of using chemicals for modification and regeneration processes should be minimized to maintain the eco-friendly nature of these adsorbents.

*Selective adsorption and competition:* In wastewater containing a complex mixture of various dyes and other pollutants, lignocellulosic materials may exhibit selective adsorption, preferentially adsorbing certain dye molecules over others. This selectivity can complicate the treatment process, requiring tailored approaches for different wastewater compositions.

Addressing these limitations and challenges is essential for advancing the use of lignocellulosic biomass materials in textile dye removal. Continued research and innovation are needed to improve the adsorption capacity, stability, regeneration efficiency, and economic feasibility of these natural adsorbents.

## 5. Future Scope and Considerations 

The potential of lignocellulosic biomass materials as adsorbents for textile dye removal presents promising avenues for future exploration and innovation. One such direction involves the development of hybrid composites and surface functionalization techniques. Researchers can enhance their adsorption effectiveness by combining lignocellulosic materials with other substances or modifying their surfaces, thereby improving water purification processes.

Moreover, there is ample room for the further optimization of process parameters to create dye removal systems that are not only efficient but also reliable, scalable, and economically viable. This optimization can lead to the development of practical solutions that can be implemented on a larger scale, addressing the pressing need for effective wastewater treatment in the textile industry.

Delving deeper into lignocellulosic biomass-based adsorption’s kinetic and mechanistic aspects opens doors to better treatment process design and more accurate modelling. Understanding the intricacies of how these materials interact with dyes in water can facilitate the creation of more efficient and tailored purification systems, ultimately contributing to improved water quality and environmental sustainability.

Exploring the use of lignocellulosic biomass materials across a broader spectrum of textile colours and their compatibility with other water treatment technologies offers a comprehensive approach to addressing water pollution challenges. By examining their performance with various dye types and in combination with other treatment methods, researchers can identify synergies and optimize overall purification processes.

Additionally, investigating regeneration protocols and conducting techno-economic evaluations are crucial steps in determining the long-term sustainability and marketability of lignocellulosic biomass materials in the wastewater treatment sector of the textile industry. Understanding how these materials can be regenerated and assessing their cost-effectiveness will inform decisions regarding their widespread adoption and commercial viability.

In conclusion, exploring lignocellulosic biomass materials holds significant potential for mitigating environmental issues and promoting eco-friendly materials in water purification procedures. Continued research and innovation in this field can lead to the development of efficient, sustainable, and economically viable solutions that benefit both industry and the environment.

## 6. Conclusions

The global agricultural industry produces residues and lignocellulosic materials, including cellulose, hemicellulose, and lignin. Burning these residues poses a considerable threat to air quality and human health. Cellulose provides structural strength to plants, while hemicellulose and lignin contribute to stiffness and rigidity, respectively. This review focuses on utilizing lignocellulosic biomass as adsorbents for removing textile dyes from water, including Congo Red, Reactive Black 5, Direct Yellow 12, Crystal Violet, Malachite Green, Acid Yellow 99, and others. It thoroughly examines the impact of various adsorption parameters on efficiency, such as adsorption isotherms, kinetics, and thermodynamics, to elucidate the adsorption mechanism. Additionally, the study explores desorption processes to assess adsorbents’ reusability. Understanding their properties and optimizing adsorption processes can effectively mitigate water pollution, remove harmful dyes, and contribute to environmental sustainability. This review offers valuable insights into leveraging lignocellulosic biomass materials for water remediation.

## Figures and Tables

**Table 1:** Effect of initial dye concentration on adsorption capacity and dye removal (%) w.r.t. adsorbent and type of dye.

Adsorbent	Type of Dye	Conc. (mg/L)	Reaction Time (min)	Adsorption Capacity (mg/g)	Dye Removal (%)	Source
*Algerian Palygorskite*	(MB)	3–30	5	2.5–10	up to 97%	[85]
*Haloxylon Recurvum* Plant	Acid Brown	10–60	180	2.8–10	--	[82]
Fava Bean Peels	(MB)	3.6–100	70	--	80–95	[86]
Fava Bean Peels	(MB)	3.6–100	70	--	70–90	[86]
Corn Silk	Reactive Blue 19	10–500	10–60	2–71	--	[87]
Corn Silk	Reactive Red 128	10–500	10–60	2–63	--	[87]
Spent Tea Leaves	Reactive Black 5	50–100	5–200	24.8–6.7	99–43	[88]
*Citrus Limetta* Peel	Malachite Green	5–25	10–60	0.17–4.7	97–95	[89]
Mango Stone Biocomposite	Crystal Violet	20–50	60	25–352	--	[90]
*Moringa Oleifera* Seed	Reactive Red 120	10–100	30	18.5–174	--	[91]
Olive Leaves Powder	Crystal Violet	10–100	5–70	5–45	--	[92]

**Table 2:** Effect of adsorbent dosage on adsorption capacity and dye removal (%) w.r.t. adsorbent and type of dye.

Type of Dye	Adsorbent	Adsorbent Dosage	Adsorption Capacity (mg/g)	Dye Removal (%)	Source
(MB)	Walnut Shell	0.5–2 g/L	179–48	--	[99]
(MB)	Alginate/Rice Husk Bio Composite	0.1–1 g	338–145	15–89	[100]
Congo Red	Raw Petroleum Coke	4–24 g/L	--	10–60	[101]
Reactive Red 198	Municipal Solid Waste Compost Ash	0.5–2 g/L	--	79–93	[102]
Cationic blue 41	Mucilage Of Salvia Seed	0.5–4 g/L	34.2–6.74	34.2–54	[103]
Remazol Brilliant Violet 5R	Eggshell	0.5–2.5 g	2.9–0.75	74.6–93.8	[104]
Remazol Brilliant Violet 5R	Calcined Eggshell	0.5–2 g	3.5–0.96	89.8–96.3	[105]

**Table 3:** Effect of adsorbent size on dye adsorption capacity and dye removal (%) w.r.t. adsorbent and type of dye.

Type of Dye	Adsorbent	Adsorbent Size	Adsorption Capacity (mg/g)	Dye Removal (%)	Source
Congo Red	Cabbage Waste Powder	150–300 to 360–4750 µm	--	76–8	[108]
Reactive Black 5	Macadamia Seed Husks	150–300 to 2360–4750 µm	--	99–33	[109]
Direct Yellow 12	Coconut Shell Activated Carbon	50, 75, 107 µm	5.5–4.5–3.5	--	[107]
Crystal Violet	Coffee Husks	0.15–0.3 to 2.36–4.75 mm	--	96–90	[110]
(MB)	Biopolymer-Based Nanocomposite	177–250 to 400–840	--	99–86	[111]

**Table 4:** Raw adsorbents based on lignocellulosic biomass materials for dyes.

Adsorbent	Type of Dye	Monolayer Adsorption Capacity (mg/g)	Source
Sugarcane Bagasse	(MB)	1.83	[44]
Rice Husk		40.6	[132]
Brewers Spent Grain		13	[133]
Orange Albedo		70.30	[83]
*Luffa Cylindrica*		49	[42]
Walnut Shell Powder		51.5	[34]
Orange Albedo		70.30	[83]
Banana Peel Powder	Reactive Blue 5	49.2	[134]
Psyllium Seed Powder	Reactive Orange 16	206	[135]
Egg Shell	Direct Blue 78	13	[47]
Mango Leaf Powder	Acid Yellow 99	708	[45]
Mushroom Waste	Direct Red 5B, Direct Black 22, Direct Black 71, and Reactive Black 5	18, 15.4, 20.1, and 14.6	[96]
Oak Saw Dust	Acid Blue 25	29.5	[136]
Walnut Shell	Acid Blue 25	36.98	[136]
Fly Ash	Reactive Black 5, Reactive Red 23, and Reactive Blue 171	4.3, 2.1, and 1.8	[137,138]
Tea Waste	Acid Green 25	123.46	[46]
Cashew Nutshell	Acid Green 25	76.34	[46]
*Rhizopus Arrhizal*	Reactive Red 4, Reactive Blue 19, and Reactive Orange16	150, 90, and 190	[139]

**Table 5:** Various biomass adsorbents studied for regeneration studies.

Type of Dye	Adsorbent	Desorption Agents	Results of Desorption	Source
Direct Orange 26, Direct Red 31, Direct Blue 67, Ever Direct Orange	Modified Rice Husk	H_2_O, NaOH, Na_2_CO_3_	62.8 mg/g, 80.5 mg/g, 53.7 mg/g, 75.3 mg/g After 10 cycles, adsorption capacity decreased by 17%	[140]
Acid Violet 17	Activated Pistachio Shell	NaCl, HCl, NaOH, CH_3_COOH	Desorption efficiency decreases from 94.6% (1st cycle) to 75.8% (3rd cycle)	[97]
(MB)	Black And Green Olive Stones	Acetic acid and ethanol	Desorption efficiency for black olive stone (92.5%) and green olive stone (88.1%)	[95]
(MB)	Modified Orange Tree Sawdust	DI water, NaCl	16.5% (using water), 58.6% (using NaCl)	[141]
Direct Blue 78	Egg Shell	NaOH	Desorption efficiency increases from 21% (1st cycle) to ~58% (3rd cycle)	[47]
Acid Yellow 99	Mango Leaf Powder	NaOH, NaCl	97% (using NaOH), 58.5% (using NaCl)	[45]
*Dioscorea Opposita Thunb. (DOT)*	Indigo Carmine	NaOH	93% desorption efficiency	[142]

## Data Availability

Not applicable.
